# Robust estimation of millisecond timescale synchrony under nonstationary conditions and its physiological interpretation

**DOI:** 10.1186/1471-2202-16-S1-P4

**Published:** 2015-12-18

**Authors:** Jonathan Platkiewicz, Kamran Diba, Pascale Quilichini, György Buzsaki, Asohan Amarasingham

**Affiliations:** 1 Department of Mathematics, City College, City University of New York, New York, NY 10031, USA; 2 Department of Psychology, University of Wisconsin-Milwaukee, Milwaukee, WI 53201, USA; 3 Aix Marseille Université, Institut des Neurosciences des Systèmes, Marseille, France; 4 Inserm, UMR_S 1106, 27 Bd Jean Moulin, 13385 Marseille Cedex 5, France; 5 Neuroscience Institute, New York University, New York, NY 10016, USA

## 

Motivated by the observation of millisecond-timescale synchrony among hippocampal neurons in high-density extracellular recordings of freely moving rats [1], we developed and applied a model for detecting synchrony under such conditions. We calibrated the model using data of a putative monosynaptic connection, obtained in paired extracellular-intracellular recordings in anesthetized rats [2].

There has been a great deal of interest in detecting synchrony, and more generally fine-temporal correlation, in the firing activities of pairs of neurons. In certain settings, a common question is whether this fine-temporal structure is supposed to reflect either a direct synaptic connection between these cells ("the monosynaptic connection hypothesis"), or a larger network organization in which these cells are embedded ("the common modulation hypothesis"). Here, we studied a specific related problem: what is a proper estimate of the amount of synchrony between two spike trains?

We modeled the problem based on a technique we call "separation of time scale" model. Among synchronous spikes, a spike can belong either to a fast timescale process, or to a slow timescale process, called background activity; non-synchronous spikes belong to background activity. The definition of timescale for these processes is based on conditional modeling tools, as developed in previous studies [3], and is designed to accommodate even extreme forms of nonstationarity in the underlying spike processes. Under this framework, we can either test the hypothesis that the activities of these two cells are uncorrelated on a fine-timescale, or estimate the number of synchronous spikes due to fine-timescale processes. It was argued however that, in the context of related models, resulting statistical hypothesis tests have low power and estimators of synchrony are biased [4]. An alternative method was proposed and observed to offer a high power test and unbiased estimation in numerical simulations. We studied this alternative approach and framed it within our separation-of-timescale model. We develop hypothesis tests and unbiased estimators and interpret and clarify previous results in this context. We applied the developed method to large-scale recordings of neuronal populations in the cornu ammonis 1 (CA1) and CA3 regions of the hippocampus of freely moving rats [1]. Finally, we also simulated a simple biophysical model of a monosynaptic connection, and demonstrated the relevance of our method in this mechanistic context.

Nevertheless, this method is based solely on an analysis of spike trains, which give a restricted representation of a cell's `true' activity and result from numerous steps of statistical inference (e.g. spike sorting). Any conclusion based on such analysis should be therefore trusted with caution. We examined an experimental dataset where a fine-temporal structure was identified using paired extracellular-intracellular in vivo recordings, in the enthorinal cortex of the anaesthetized rat [2]. This dataset allowed us to calibrate our spike train analysis method (i.e. supervised learning), and to advance existing methodologies for identifying functional connectivity from in vivo recording.
